# Direct observation of ideal electromagnetic fluids

**DOI:** 10.1038/s41467-022-32187-2

**Published:** 2022-08-12

**Authors:** Hao Li, Ziheng Zhou, Wangyu Sun, Michaël Lobet, Nader Engheta, Iñigo Liberal, Yue Li

**Affiliations:** 1 Department of Electronic Engineering, Beijing National Research Center for Information Science and Technology, Tsinghua University, Beijing, 100084 China; 2 Department of Physics and Namur Institute of Structured Materials, University of Namur, Rue de Bruxelles 61, 5000 Namur, Belgium; 3 Department of Electrical and Systems Engineering, University of Pennsylvania, Philadelphia, PA 19104 USA; 4 Department of Electrical and Electronic Engineering, Institute of Smart Cities (ISC), Public University of Navarre (UPNA), Pamplona, 31006 Spain

**Keywords:** Metamaterials, Electrical and electronic engineering, Metamaterials

## Abstract

Near-zero-index (NZI) media have been theoretically identified as media where electromagnetic radiations behave like ideal electromagnetic fluids. Within NZI media, the electromagnetic power flow obeys equations similar to those of motion for the velocity field in an ideal fluid, so that optical turbulence is intrinsically inhibited. Here, we experimentally observe the electromagnetic power flow distribution of such an ideal electromagnetic fluid propagating within a cutoff waveguide by a semi-analytical reconstruction technique. This technique provides direct proof of the inhibition of electromagnetic vorticity at the NZI frequency, even in the presence of complex obstacles and topological changes in the waveguide. Phase uniformity and spatially-static field distributions, essential characteristics of NZI materials, are also observed. Measurement of the same structure outside the NZI frequency range reveals existence of vortices in the power flow, as expected for conventional optical systems. Therefore, our results provide an important step forward in the development of ideal electromagnetic fluids, and introduce a tool to explore the subwavelength behavior of NZI media including fully vectorial and phase information.

## Abstract (web summary)

This paper reports an experimental observation of irrotational, inviscid, and incompressible electromagnetic power flow within an epsilon-near-zero medium, exhibiting an analogy to an ideal fluid.

## Introduction

Recent years have witnessed a surge of interest in the counterintuitive physical phenomena taking place in near-zero index (NZI) media^1^. Due to the infinitely stretched wavelength and spatially-static fields, electrodynamics inside NZI media lead to a series of physical effects where some observables are independent of the geometry of the system. Popular examples include supercoupling^2–6^, deformable electromagnetic resonators^7^, photonic doping^8–11^, and the enhancement of the spatial coherence of thermal fields^12^. These exotic physics also empower numerous technological applications in a wide spectrum from radio to optical frequencies, including antennas^13–16^, lenses^17–19^, and components with boosted optical nonlinearities^20–24^. The underlying mechanism is attributed to the decoupling between spatial (wavenumber) and temporal (frequency) variations of electromagnetic fields, leading to spatially static, but temporally dynamics field distributions^25^. For experimental verifications of these properties, the scattering parameters are measured with respect to either spectral or angular variations under deformations of NZI media’s geometries^26–28^. However, the local and/or subwavelength details of the field distributions within NZI media have been much less studied. Exceptions include the direct observation of standing waves^29^ and the position-independence of cathodoluminescence within NZI waveguides^30^. In both cases, the experiment retrieves a scalar image of the amplitude along a straight waveguide. However, there is no characterization of the vectorial character of the field distributions within the NZI media, i.e., phase and amplitude information, within nontrivial geometries.

Local and subwavelength details of the field distributions inside NZI media present rich physics. For example, the local electromagnetic power flow—represented by the Poynting vector field—inside NZI media is mathematically equivalent to the velocity field occurring in an ideal fluid^31^. As a consequence, optical turbulence is intrinsically inhibited in NZI media, suppressing any vorticity in the power flow. Light propagation within NZI media can be understood as an electromagnetic ideal fluid, the electromagnetic equivalent of an inviscid, incompressible, and irrotational fluid.

**Fig. 1:**
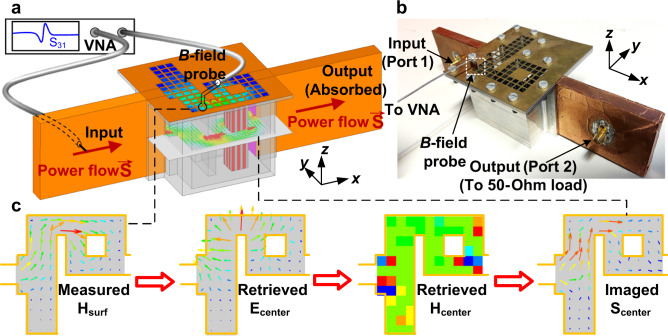
Conceptual sketch of the experimental system and data processing method. **a** The experimental equipment includes an epsilon-near-zero (ENZ) waveguide for ideal fluid power flow, input and output waveguides, a *B*-field probe, and a vector network analyzer (VNA). The central plane of the waveguide shows the Poynting vectors obtained by numerical simulation. **b** The photo of the experimental platform, which contains the waveguide channel and the *B*-field probe. **c** The data processing method for experimentally observing the power flow on the center plane.

In this paper, we report an experimental demonstration of ideal electromagnetic fluids at microwave frequencies using a dispersive rectangular waveguide at its cutoff frequency (Fig. 1), acting as an epsilon-near-zero (ENZ) structure, which is a specific kind of NZI medium. Such dispersive waveguides feature smaller losses than actual ENZ materials^32–34^. Using this platform, we are able to build nontrivial geometries, including deformation and blockage of the direct propagation path between input and output ports, which favor optical turbulence. The topology of the geometry is further modified by introducing dielectric particles in such waveguides. Moreover, we develop a dedicated retrieval procedure that enables the direct mapping of the fields inside the waveguide with fully vectorial information, encompassing phase and amplitude, based on only surface measurements. Such a method has negligible interferences to the original field within the cavity and is feasible to be used in various photonics applications. The experimental results confirm that under the ENZ condition, neither the severe deformations of channels nor the existence of inclusions induces vorticities to the electromagnetic power flow, while vortexes are observed when operating away from the ENZ condition. Our results experimentally demonstrate the analogy between an ideal fluid and electromagnetic power flow in NZI media. This conclusion is an important step forward in the development of ideal electromagnetic fluids, provides physical insight into the supercoupling effect, and opens perspectives for applications within the field of light propagation within waveguides.

## Results

### The power flow in waveguide-emulated ENZ media

As demonstrated theoretically^31^, the distribution of the Poynting vector field in two-dimensional (2D) ENZ media is mathematically equivalent to the velocity vector field of an ideal fluid, which is inviscid, incompressible, and irrotational. These three critical properties come along with three different vector calculus equations. For the viscosity elimination, we have **n** · **S**_**R**_ = **0** on the boundary of the conduit, where **n** is a unit vector normal to the boundary and **S**_R_ is the real part of the Poynting vector. A physical insight of this equation is that no power goes out of the ENZ medium, i.e., the boundary is impenetrable except for the input and output ports. An incompressible power flow is characterized by a divergenceless vector field, i.e., ${{{{{\boldsymbol{\nabla }}}}}}\cdot {{{{{{\bf{S}}}}}}}_{{{{{{\rm{R}}}}}}}=0$, which is found for any lossless material. The former two properties are satisfied for any electromagnetic lossless medium. However, the most critical one is that the Poynting vector field is irrotational, i.e., ${{{{{\rm{\nabla }}}}}}\times {{{{{{\bf{S}}}}}}}_{{{{{{\rm{R}}}}}}}={{{{{\bf{0}}}}}}$. It means that the electromagnetic power smoothly flows around the obstacles and dielectric inclusions without forming any vortexes. This condition can only be met in NZI media^31^.

To achieve a low-loss ENZ medium, we use a waveguide structure as shown in Fig. 1a. First, the input and output waveguides are composed of a Teflon brick coated with copper foils (orange—Fig. 1a). The ports are numbered as Port 1 and 2, respectively. Second, to mimic an ENZ medium, we use an air-filled aluminum case of height $h \sim \lambda /2$ (transparent gray—Fig. 1a). Detailed dimensions are provided in Fig. S1 (See Supplementary Information). A photo of the fabricated waveguide platform is shown in Fig. 1b. The detailed fabrication and assembling techniques are depicted in Materials and Methods. To numerically investigate the irrotational feature, we performed a simulation of the electromagnetic field distributions using ANSYS HFSS^®^ 18 within the waveguide (see Materials and Methods). We plot the Poynting vectors located on the central plane inside the sketch of the structure shown in Fig. 1a. One can see that there are no vortexes of the Poynting vector within the ENZ region. To further investigate the lack of vorticity in the designed waveguide structure, a comparison has been made between an ideal 2D ENZ medium and the proposed structure in Fig. S2 (See Supplementary Information). The numerical simulations confirm that the fields on the central plane of the waveguide provide an exact replica of the field distribution in an ideal 2D ENZ medium.

### Simulation and experimental verifications

The designed waveguide structure, which effectively represents an ENZ medium, can be used to experimentally measure the electromagnetic fields and to observe the ideal electromagnetic fluid property. However, such an enclosed waveguide structure does not allow for the measurement of internal electromagnetic fields and its subwavelength details. Moreover, the field distribution in the waveguide is only equivalent to an ENZ medium at its center plane. Furthermore, introducing a probe to measure the field inside the waveguide would result in a strong perturbation of the field distribution. This is why most previous experiments on ENZ waveguides have mostly focused on measuring the scattering (reflection/transmission) parameters^26–28^. To overcome this challenge, in this section, we introduce a reconstruction technique that allows for direct observation of the power flow in the center plane of the waveguide. The general outline of the procedure is shown in Fig. 1c and can be summarized as follows. We first measure the tangential components of the magnetic field on the top surface, from which we then retrieve the electric field in the central plane. From this information, we deduce the magnetic field in this central plane and finally calculate the Poynting vector in this plane (see mathematical development below). In fact, our procedure allows for the mapping of field distributions with complete vectorial information, i.e., acquiring both amplitude and phase.

To experimentally measure the magnetic field on the top surface, we need to slightly modify the geometry of the structure. In particular, instead of a full metallic cover, the top of the cavity is shielded by a metal grid cover on which identical square holes are etched, enabling the measurement of discretized surface magnetic fields (top part of Fig. 1a). The size and number of each hole are related to the resolution of the measurement. In other words, enhancing the number of holes may lead to a finer result. Since the holes are electrically small, the metal grid cover still effectively behaves as a conductive boundary condition and the electromagnetic energy is confined within the cavity. We numerically verified the validity of this modification by comparing both transmission spectra and the power flows of the structures using a full metal cover and the perforated metal grid in Fig. S3 (See Supplementary Information). This structure supports an almost total transmission at the frequency of 3.06 GHz where the ENZ medium exhibits a near-zero effective permeability^8,10^. The similarity of the field pattern allows us to conclude that the top metallic grid allows for the measurement of the magnetic field on the top surface of the waveguide while having a negligible impact on the field distributions in the center plane. Ideally, if we want to measure the fields in the central plane, introducing an electric or magnetic field probe directly into the center of the cavity would disturb the internal field distribution. Actually, there has not been a method reported by previous literature that completes the measurement of electromagnetic fields without obvious interferences of the original field. Instead, we use an alternative measurement method in order to avoid any major field perturbation. Specifically, we develop an electrically small metal loop connected to a coaxial cable as a *B*-field probe to measure the complex-valued tangential components of the magnetic fields on the top surface. This probe is inserted into the holes of the metallic grid. The coaxial port connected to the probe is denoted as Port 3. When measuring the magnetic fields, the output waveguide is connected to a 50-Ohm load for absorption and a vector network analyzer is used to measure the transmission coefficient from the input waveguide to the probe, denoted as S_31_. The measured values of the S_31_ coefficient in different holes and different orientations provide us with a map of the normalized magnitude and phase distribution of magnetic fields in both *x* and *y* directions. To validate the quantitative values, we take two specific lines within the ENZ region as examples and investigate the relations between the simulated magnitudes of fields, the simulated values of S_31_, and the measured values of S_31_. The results are shown in Fig. S6 (See Supplementary Information), demonstrating the consistency of the proposed method when those values are normalized to their maxima. Since the magnitude of S_31_ is usually lower than −20 dB, indicating that no larger than 1% of the power is coupled to the probe, it can be concluded that the interferences to the field inside the cavity is small enough to be neglected.

With the measured magnetic fields on the top surface (and the fact that the tangential components of the electric field are zero on this plane), we can retrieve the electromagnetic fields at any location in this cavity. Since the width of the waveguide and the cavity is uniform and smaller than the wavelength, we can ensure that only the TE_10_ mode exists in the cavity while higher ordered modes decay very rapidly in the input waveguide. Under single TE_10_ mode excitation, we develop an analytical retrieval procedure. Figure 1c shows the retrieval procedure starting from the measured magnetic field on the surface and finally resulting in the image of the power flow on the central plane. This procedure is analytically justified as follows. Starting from the measured magnetic fields on the surface at $z=h/2$, we can retrieve the electric fields on the center plane *z* = 0

1 $${{{{{\bf{E}}}}}}(x,y,0)=\frac{i\omega {\mu }_{0}h}{\pi }{{{{{\bf{H}}}}}}(x,y,\frac{h}{2})\times \hat{{{{{{\bf{z}}}}}}}.$$

A time-convention exp(-*iωt*) is assumed and omitted hereafter. Noting that the central plane is equivalent to a 2D medium with a Drude dispersive permittivity and vacuum permeability *μ*_0_ and using the Faraday law, the magnetic fields on the center plane is retrieved by calculating the curl of the electric field:

2 $${{{{{\bf{H}}}}}}(x,y,0)=\frac{1}{i\omega {\mu }_{0}}{{{{{{\boldsymbol{\nabla }}}}}}}_{xy}\times {{{{{\bf{E}}}}}}(x,y,0).$$

Where **∇**_*xy*_ is the 2D nabla operator. For this time-harmonic field, the real part of the Poynting vector, which represents the time-averaged power flow on the central plane, is thus directly calculated from the fields by

3 $${{{{{{\bf{S}}}}}}}_{{{{{{\rm{R}}}}}}}(x,y,0)=\frac{1}{2}{{{{\mathrm{Re}}}}}[{{{{{\bf{E}}}}}}(x,y,0)\times {{{{{\bf{H}}}}}}(x,y,0)].$$

By substituting the results of Eq. (1) and Eq. (2) into Eq. (3), we can obtain the power flow distribution on the central plane (*z* = 0) from the measured magnetic fields on the $z=h/2$ plane. Notice that both the electromagnetic fields and Poynting vectors distribute in a two-dimensional medium, we believe that the full vectorial characterization is included in the magnitude and phase of two orthogonal components. A detailed derivation of Eq. (1) is provided in Supplementary Note 3 (See Supplementary Information).

### Experimental results

**Fig. 2:**
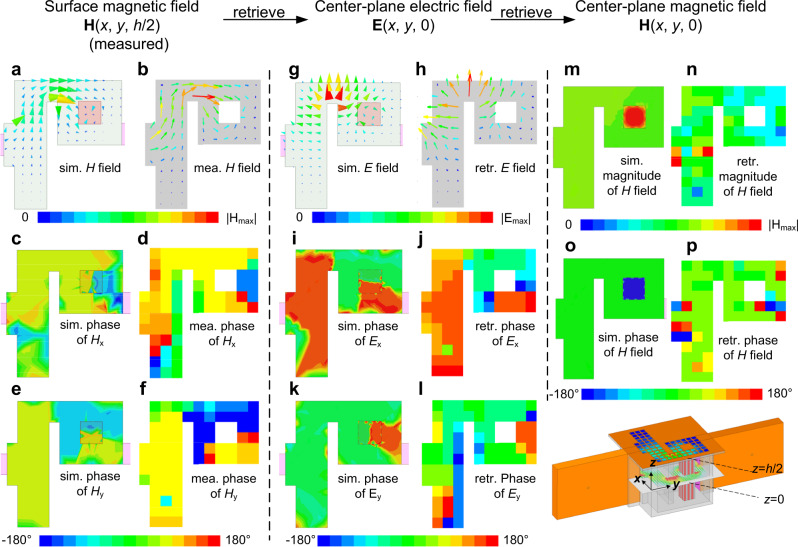
Reconstruction process and comparison between simulated and measured or imaged field distributions in each step. **a**–**f** simulated and measured vector and phase distribution of the surface magnetic field, **g**–**l** simulated and retrieved vector and phase distribution of the electric field on the center plane, **m**–**p** magnitude and phase of simulated and retrieved magnetic field’s *z* component on the center plane.

The experiment is launched on the platform shown in Fig. 1b and the retrieval method is applied to observe the absence of vorticity in the power flow within the ENZ waveguide using the system shown in Fig. S4 (See Supplementary Information). Two specific frequencies of 3.06 GHz and 3.9 GHz are investigated. According to the numerical simulations, the waveguide structure shows an ENZ response at approximately 3.06 GHz (see Figs. S3 and S5 (See Supplementary Information)). Since the effective permittivity of the waveguide can be described using the Drude model *ε*_eff_ = *ε*_0_(1 − *ω*_0_^2^/*ω*^2^) where ω_0_ is the cutoff frequency of the TE_10_ mode^4^, the structure acts as a conventional medium with an effective relative permittivity of 0.4 at 3.9 GHz. In addition, the designed waveguide can be modified by introducing a dielectric dopant in order to enhance the field magnitude inside the waveguide, obviously stronger than that for the ENZ medium without a dopant. Indeed, dielectric particles immersed in ENZ media act as photonic dopants that modify its effective permeability, while maintaining a near-zero permittivity^8^. A maximized transmission is then obtained by tuning its effective permeability to zero since theoretically perfect transmission is obtained for doped ENZ media. As anticipated, such supercoupling takes place for any arbitrary geometry^8^. Therefore, by tuning the dielectric particle characteristics so that the effective permeability approaches zero for impedance matching, the magnitude of the Poynting vector field can be greatly enhanced. Moreover, the inclusion of dielectric particles changes the topology of the waveguide. It contains obstacles that are qualitatively different from deformations of the waveguide walls, and consequently, the vorticity of the power flow can be investigated under a richer configuration showing topology changes. Here four different configurations can be investigated using the designed waveguide: the doped or undoped waveguide at two different frequencies (3.06 GHz—the ENZ frequency—and 3.9 GHz—frequency showing a conventional positive-permittivity material). In the main text, for the sake of brevity, we only present the measured and retrieved electromagnetic field results of the doped structure at 3.06 GHz, mimicking the doped ENZ material (Fig. 2) in terms of scattering properties. The word “retrieve” means analytical calculations performed to the directly measured data according to Eqs. (1)–(3). Fields in the other three situations are shown in Figs. S8–S10 (See Supplementary Information). Note that fields are only measured within the hollow waveguide because the probe cannot be inserted into the dopant. The simulated and measured magnetic fields on the top surface are plotted in the left panel of Fig. 2 (Figs. 2a–f). Both the vector and phase distributions of simulations and measurement match very well, except for the locations where the field is too weak. The measurement shown in Fig. 2 are further validated by comparing the simulated and measured S_31_ (see Fig. S7 (See Supplementary Information)).

**Fig. 3:**
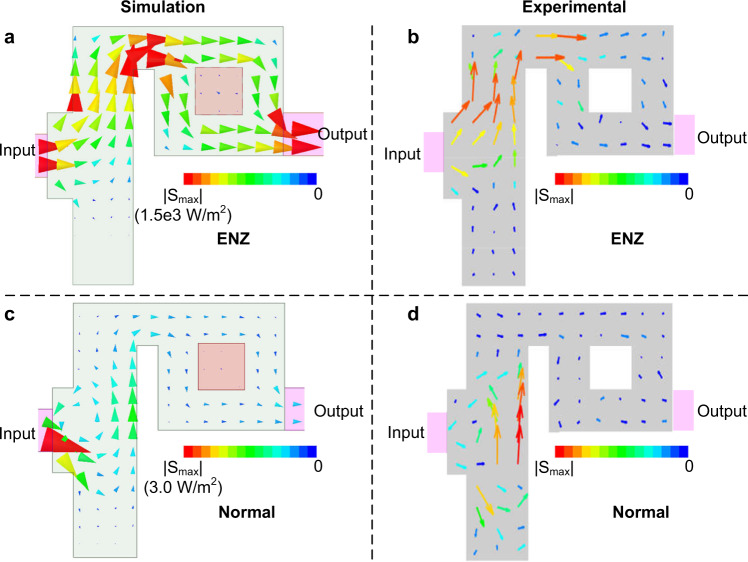
Simulated and experimental observation of the power flow within doped ENZ and normal media. **a**, **b** the simulated and experimentally imaged power flow of the doped ENZ medium at 3.06 GHz, **c**, **d** the simulated and experimentally reconstructed power flow of the normal medium with the same dielectric particle but the operating frequency at 3.9 GHz.

As explained above, this measurement allows us to retrieve the electric fields on the center plane (central panel of Fig. 2 also with simulated results, Fig. 2g–l). Both the simulated and measured ones yield that the electric field distribution resembles a spatially electrostatic field (while it is temporally dynamic). In particular, the electric fields are perpendicular to the electrostatic potential lines between the waveguide walls^29^, with the fields being concentrated at the corners of the waveguide deformations. This result provides experimental proof of the spatially-static field distributions taking place in doped ENZ media, including its fully vectorial characterization. Furthermore, the magnitude and the phase of the *z* component of the magnetic field in the central plane is retrieved by calculating the curl of the electric field as reported in the right panel of Fig. 2 (Fig. 2m–p). Our results provide a clear picture of the magnitude and phase uniformity of the magnetic field, one of the main characteristics of 2D doped ENZ media^31^. Most previous works have indirectly characterized geometry-invariant phenomena on NZI media via the measurement of scattering parameters^26–28^. By contrast, here, our retrieval technique provides direct observation with fully vectorial and phase information at the field distribution level, confirming the existence of spatially static but temporally dynamic field distributions. The same technique could be applied to a large number of wave phenomena in NZI media. Although some inaccuracies might be found in Fig. 2d, l, p near the PEC wall, this inconstancy in the phase measurement does not impede the power flow reconstruction, as shown in Fig. 3, since the intensity of the measured electromagnetic field is low.

Now, we have everything at hand to investigate the Poynting vector on the central plane, demonstrating the absence of vorticity. First, this phenomenon is numerically exhibited in the proposed configurations as shown in Figs. 3a–c. Then, based on the retrieved electromagnetic fields on the center plane (*z* = 0), we generate an experimental map of the power flow **S**_R_ (*x*, *y*, 0) in this plane and plot them for both ENZ frequencies at 3.06 (Fig. 3b) and 3.9 GHz (Fig. 3d). Experiment and theory show good agreement except small inaccuracies where the values of the measured Poynting vector are small. The experimental map depicts some small Poynting vectors toward the dopant more obvious than the simulations because of a higher dielectric loss than simulated ones. The vortex-free nature of the power flow is evidenced in the results, both for simulation and retrieved measurement (Fig. 3a, b, where we have the ENZ behavior). The analogy with an ideal fluid is further validated by noting that the existence of dielectric dopant does not generate any vorticities in the Poynting vector field. It emphasizes that this electromagnetic fluid property is protected at the local level, i.e., it does not depend on the geometry and/or topology of the system. Moreover, it is shown that the power flow distribution circumvents the obstacle (Fig. 3a, b). Despite the fact that the obstacle consists of a penetrable dielectric, with nonzero fields on its interior, it behaves as an opaque obstacle in terms of the power flow. Furthermore, the Poynting vector has an increased intensity within the narrow channels above the obstacle, similar to the expected behavior in fluid mechanics theory, where a fluid accelerates when flowing through a narrow pipe^35^. Those phenomena are fully consistent with the theory introduced in the previous work^31^.

For the sake of comparison, the power flow in a normal medium (relative permittivity of 0.4 @3.9 GHz) is also investigated using the same numerical and experimental conditions. Although the permittivity is below unity, the value is sufficiently high to observe a completely different behavior for the Poynting vector field. Specifically, a strong vortex in the power flow is predicted by the numerical simulations in Fig. 3c, and experimentally observed in Fig. 3d. The electromagnetic fields retrieved at each stage to image Fig. 3d are presented in Fig. S8 (See Supplementary Information). This vorticity shows that a major part of the impinging power is reflected back into the input port by the obstacle so that the transmission through this channel is blocked. Our results emphasize that most electromagnetic and optical systems exhibit vortexes in the presence of obstacles, and it is a unique property of NZI, here doped ENZ, media that the power flow is protected against the creation of vortexes at a local level. Thus, NZI media are platforms where electromagnetic radiations act as ideal electromagnetic fluids, where optical turbulence is intrinsically inhibited.

**Fig. 4:**
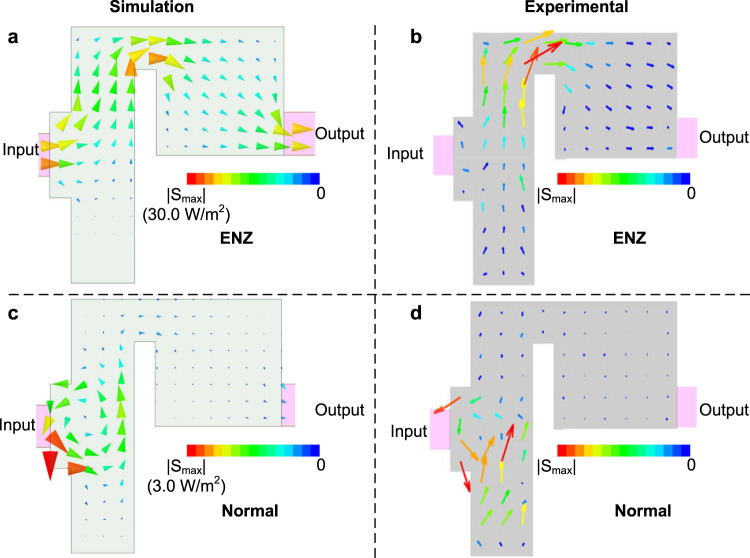
Simulated and experimentally imaged power flow within non-doped ENZ and normal media. **a**, **b** the simulated and experimentally imaged power flow of the non-doped ENZ medium, **c**, **d** the simulated and experimentally reconstructed power flow of the normal medium. The operating frequency is 3.9 GHz for this case.

The versatility of our designed platform allows us to investigate the ENZ media without the dopant. By doing so, the relative permeability of the ENZ medium is unity so that a large amount of transmission power is blocked because of impedance mismatch. However, both numerical and experimental results illustrate that this blockage for the ENZ case (with 3.06 GHz and no dopant), is intrinsically different from what we can observe in a conventional medium (frequency of 3.9 GHz where the effective permittivity of the waveguide is 0.4). To be specific, the simulated and experimentally reconstructed images of Poynting vectors are shown in Fig. 4a, b. Both simulations and experiments further demonstrate the ideal fluid property of the power flow near the conductive obstacle with or without the dielectric dopant. However, removing the dielectric dopant reduces the magnitude of the power flow by more than 17 dB and consequently, the transmittance is lower here than in the doped case since the maximums of power flow in Fig. 3a and b are 50 times higher than those in Fig. 4a, b. Vorticities of the Poynting vectors in regular media with positive-permittivity can be observed both in simulations and experiments, as illustrated in Fig. 4c, d. The major part of the electromagnetic power is blocked by the conductive obstacle inducing optical turbulence. The electromagnetic fields retrieved at each stage to reconstruct Fig. 4b, d are presented in Figs. S9 and S10 (See Supplementary Information).

## Discussion

We have designed and performed an experiment to directly map the Poynting vector field on the central plane within a rectangular waveguide, operating at its TE_10_ mode’s cutoff, acting as a two-dimensional NZI medium. Our results experimentally validate the behavior of ENZ media where electromagnetic radiations act as ideal electromagnetic fluids. The images demonstrate that the power flow in the waveguide smoothly adapts to the waveguide deformations, that the power flow is concentrated around the corners of the obstacles, and, more importantly, that the formation of vortexes is forbidden. Furthermore, these features are proven to be robust against topological deformations of the waveguide by introducing dielectric particles acting as photonic dopants. Electromagnetic ideal fluids empower a new field of multiphysics applications, including systems protected against deformations and/or mechanical perturbations, as well as optical systems inspired by fluid mechanics and airfoil theory. We believe that the experimental verification of electromagnetic fluids is an important step in the field, and it will motivate further research in systems where optical turbulence is intrinsically forbidden. Our experiment has also provided direct observation of spatially static electric field distributions, as well as magnetic fields with magnitude and phase uniformity, i.e., the two main electrodynamics characteristics of 2D ENZ media. We expect that our results will encourage a more advanced characterization of the exotic wave phenomena taking place in NZI media.

## Methods

### Numerical calculations

The numerical simulations on the 3D structure have been carried out with ANSYS HFSS^®^ 18. A 50-Ohm lumped port is used for excitations at the position where sub-miniaturized A (SMA) connectors are located. The aluminum and copper used in the model are seen as perfect electric conductor (PEC) and their Ohmic losses are neglected. In particular, the aluminum shell of the ENZ cavity is set to be PEC material and the copper covering the waveguide is simulated by applying PEC boundaries on the Teflon brick. In addition, the 2D simulations for Fig. S2 are completed using the COMSOL Multiphysics^®^ 5.5. A rectangular port has been used with the power of 1 W. The maximum size of the mesh element is 3 mm and the minimum size is 0.0285 mm.

### Experimental setup and measurements

The aluminum cavity and the brass grid cover of the experimental platform is constructed using computer numerical controlled (CNC) machining technology with a tolerance of 0.1 mm. The distribution of the holes on the cover is conformal with respect to the inner sidewalls of the case. In other words, all the holes are located over the area above the empty waveguide space. The dielectric block is composed by JJD37-6 microwave ceramic with relative permittivity of 37.0 and a loss tangent of 0.001. The input and output waveguides are made by Teflon with relative permittivity of 2.1. These components are assembled and screwed together using twelve M5 screws. The *B*-field probe is constructed using printed circuit board (PCB) technology on an FR-4 substrate with relative permittivity of 4.4 and a loss tangent of 0.02. It is soldered to one end of a semi-rigid coaxial cable, the other of which is connected to an SMA connector. The *S*-parameters are measured using a Keysight N9917A vector network analyzer with two ports. When measuring the magnetic field, these two ports are connected to the input port and the semi-rigid coaxial cable of the *B*-field probe. In this case, a 50-Ohm load is connected to Port 2. To measure the *x*-component of the magnetic field at each location, the *B*-field probe is placed in *yz* plane and perpendicular to the *x* orientation, and vice versa for the *y*-components.

## Supplementary information

Supplementary Materials

## Data Availability

All data needed to evaluate the conclusions in the paper are present in the paper and the Supplementary Information. Additional data related to this paper may be available at https://www.dropbox.com/s/vbg9dih3unt5xhw.

## Code availability

All the codes needed to process the data are provided at https://www.dropbox.com/s/vbg9dih3unt5xhw.

## References

[1] Liberal I, Engheta N (2017). Near-zero refractive index photonics. Nat. Photonics.

[2] Silveirinha M, Engheta N (2006). Tunneling of electromagnetic energy through subwavelength channels and bends using ε-near-zero materials. Phys. Rev. Lett..

[3] Ma HF, Shi JH, Cheng Q, Cui TJ (2013). Experimental verification of supercoupling and cloaking using mu-near-zero materials based on a waveguide. Appl. Phys. Lett..

[4] Edwards B, Alù A, Young ME, Silveirinha M, Engheta N (2008). Experimental verification of epsilon-near-zero metamaterial coupling and energy squeezing using a microwave waveguide. Phys. Rev. Lett..

[5] Liu R (2008). Experimental demonstration of electromagnetic tunneling through an epsilon-near-zero metamaterial at microwave frequencies. Phys. Rev. Lett..

[6] Mitrovic M, Jokanovic B, Vojnovic N (2013). Wideband tuning of the tunneling frequency in a narrowed epsilon-near-zero channel. IEEE Antennas Wirel. Propag. Lett..

[7] Liberal I, Mahmoud AM, Engheta N (2016). Geometry-invariant resonant cavities. Nat. Commun..

[8] Liberal I, Mahmoud AM, Li Y, Edwards B, Engheta N (2017). Photonic doping of epsilon-near-zero media. Science.

[9] Luo J, Liu B, Hang ZH, Lai Y (2018). Coherent perfect absorption via photonic doping of zero-index media. Laser Photonics Rev..

[10] Zhou Z (2019). Substrate-integrated photonic doping for near-zero-index devices. Nat. Commun..

[11] Zhou Z, Li Y (2021). N-port equal/unequal-split power dividers using epsilon-near-zero metamaterials. IEEE Trans. Microw. Theory Techn.

[12] Liberal I, Engheta N (2018). Manipulating thermal emission with spatially static fluctuating fields in arbitrarily shaped epsilon-near-zero bodies. Proc. Natl Acad. Sci. USA.

[13] Enoch S, Tayeb G, Sabouroux P, Guérin N, Vincent P (2002). A metamaterial for directive emission. Phys. Rev. Lett..

[14] Forati E, Hanson GW, Sievenpiper DF (2015). An epsilon-near-zero total-internal-reflection metamaterial antenna. IEEE Trans. Antennas Propag..

[15] Memarian M, Eleftheriades G (2015). Dirac leaky-wave antennas for continuous beam scanning from photonic crystals. Nat. Commun..

[16] Zhou Z, Li Y (2020). A photonic-doping-inspired SIW antenna with length-invariant operating frequency. IEEE Trans. Antennas Propag..

[17] Navarro-Cía M, Beruete M, Campillo I, Sorolla M (2011). Enhanced lens by ε and μ near-zero metamaterial boosted by extraordinary optical transmission. Phys. Rev. B.

[18] Navarro-Cía M, Beruete M, Sorolla M, Engheta N (2012). Lensing system and Fourier transformation using epsilon-near-zero metamaterials. Phys. Rev. B.

[19] Soric JC, Alù A (2015). Longitudinally independent matching and arbitrary wave patterning using ε-near-zero channels. IEEE Trans. Microw. Theory Techn..

[20] Alam MZ, De Leon I, Boyd RW (2016). Large optical nonlinearity of indium tin oxide in its epsilon-near-zero region. Science.

[21] Carnemolla EG (2018). Degenerate optical nonlinear enhancement in epsilon-near-zero transparent conducting oxides. Opt. Mater. Express.

[22] Capretti A, Wang Y, Engheta N, Negro LD (2015). Comparative study of second-harmonic generation from epsilon-near-zero indium tin oxide and titanium nitride nanolayers excited in the near-infrared spectral range. ACS Photonics.

[23] Kinsey N (2015). Epsilon-near-zero Al-doped ZnO for ultrafast switching at telecom wavelengths. Optica.

[24] Reshef O, De Leon I, Alam MZ, Boyd RW (2019). Nonlinear optical effects in epsilon-near-zero media. Nat. Rev. Mater..

[25] Ziolkowski RW (2004). Propagation in and scattering from a matched metamaterial having a zero index of refraction. Phys. Rev. E.

[26] Moitra P (2013). Realization of an all-dielectric zero-index optical metamaterial. Nat. Photonics.

[27] Li Y (2015). On-chip zero-index metamaterials. Nat. Photonics.

[28] Edwards B, Alù A, Silveirinha MG, Engheta N (2009). Experimental verification of plasmonic cloaking at microwave frequencies with metamaterials. Phys. Rev. Lett..

[29] Reshef O (2017). Direct observation of phase-free propagation in a silicon waveguide. ACS Photonics.

[30] So J, Yuan G, Soci C, Zheludev NI (2020). Enhancement of luminescence of quantum emitters in epsilon-near-zero waveguides. Appl. Phys. Lett..

[31] Liberal I, Lobet M, Li Y, Engheta N (2020). Near-zero-index media as electromagnetic ideal fluids. Proc. Natl Acad. Sci. USA.

[32] West PR (2010). Searching for better plasmonic materials. Laser Photonics Rev..

[33] Naik G, Kim J, Boltasseva A (2011). Oxides and nitrides as alternative plasmonic materials in the optical range. Opt. Mater. Express.

[34] Ordal M, Bell R, Alexander R, Long L, Querry M (1985). Optical properties of fourteen metals in the infrared and far infrared: Al, Co, Cu, Au, Fe, Pb, Mo, Ni, Pd, Pt, Ag, Ti, V, and W. Appl. Opt..

[35] White, F. M. *Fluid Mechanics* (McGraw-Hill Education, 2015).

