# Supplementary material for: Direct observation of ideal electromagnetic fluids
Source: Nat Commun. 2022 Aug 12;13:4747. doi: 10.1038/s41467-022-32187-2 (PMC9374703; doi:10.1038/s41467-022-32187-2)
Supplement: Supplementary file 1 — Supplementary Materials [file 41467_2022_32187_MOESM1_ESM.pdf]

Direct Observation of Ideal Electromagnetic Fluids

Li et al.

Supplementary Figures

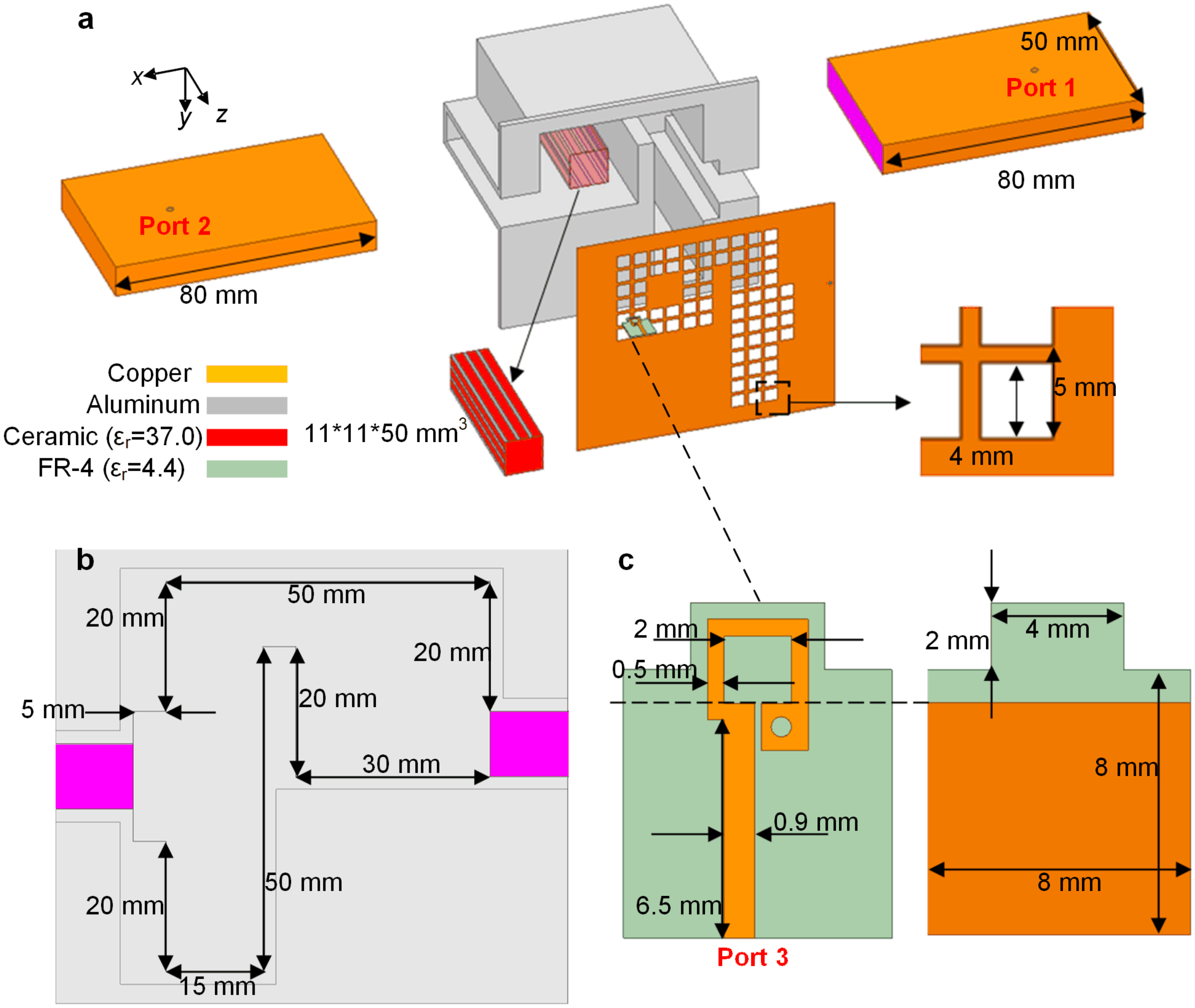

Supplementary Figure 1 | Detailed dimensions of the experimental platform: (a) the exploded view of the whole structure, **(b)** detailed geometries of the metal cavity and **(c)** detailed geometries of the *B*-field probe.

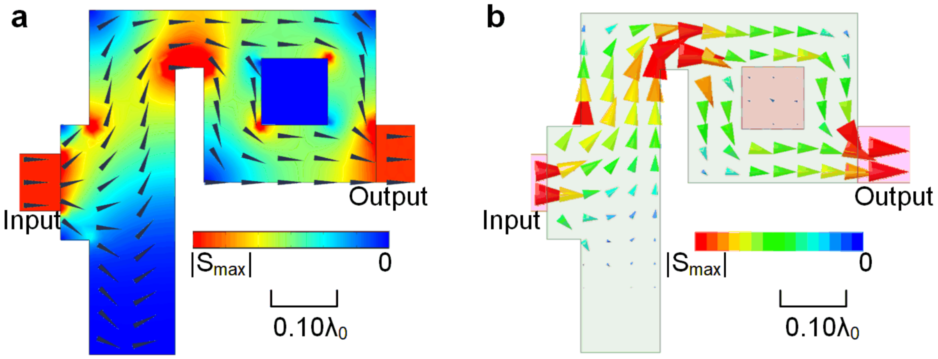

Supplementary Figure 2 | Simulated power flows in ideal and equivalent ENZ media: (a) the power flow in an ideal 2D ENZ medium with no loss. The waveguide is filled with air while the dopant's permittivity is 36.0. **(b)** the power flow on the center plane of the proposed 3D waveguide structure.

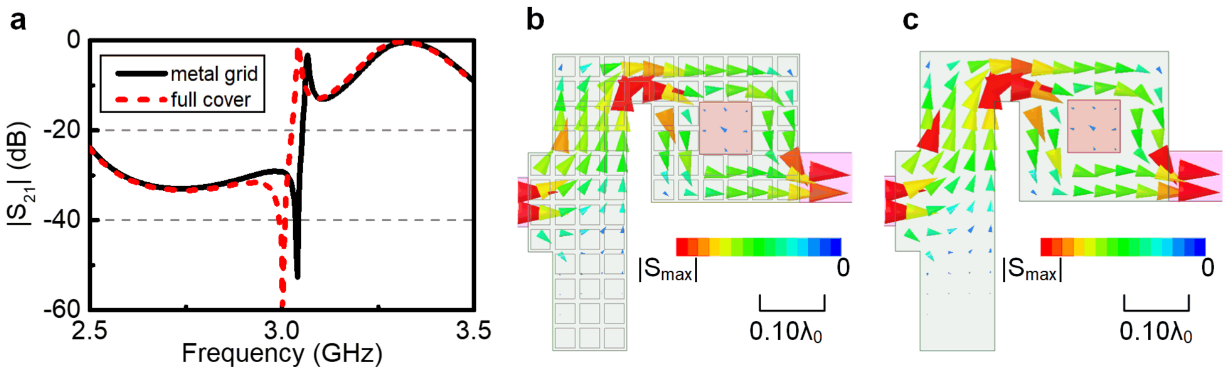

Supplementary Figure 3 | Comparison between the structure with a full metal cover and a metal grid cover: (a) Simulated transmission coefficients from Port 1 to Port 2, **(b)** and **(c)** the simulated Poynting vectors of these cases.

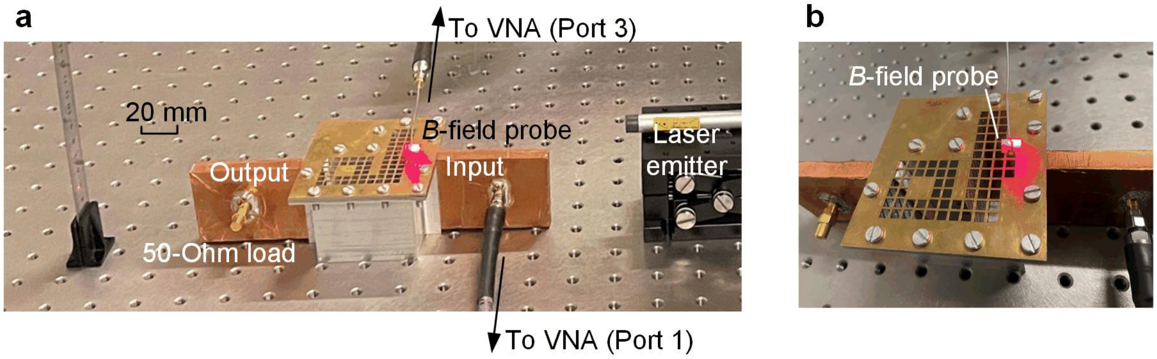

Supplementary Figure 4 | Setup of the measuring system: Photos of **(a)** the entire measuring system in which the coupling coefficient from input port to the probe is measured. **(b)** The inner structure of the waveguide section. **(c)** The detailed structure of the *B*-field probe and zoomed view of the metal loop at the end of the probe.

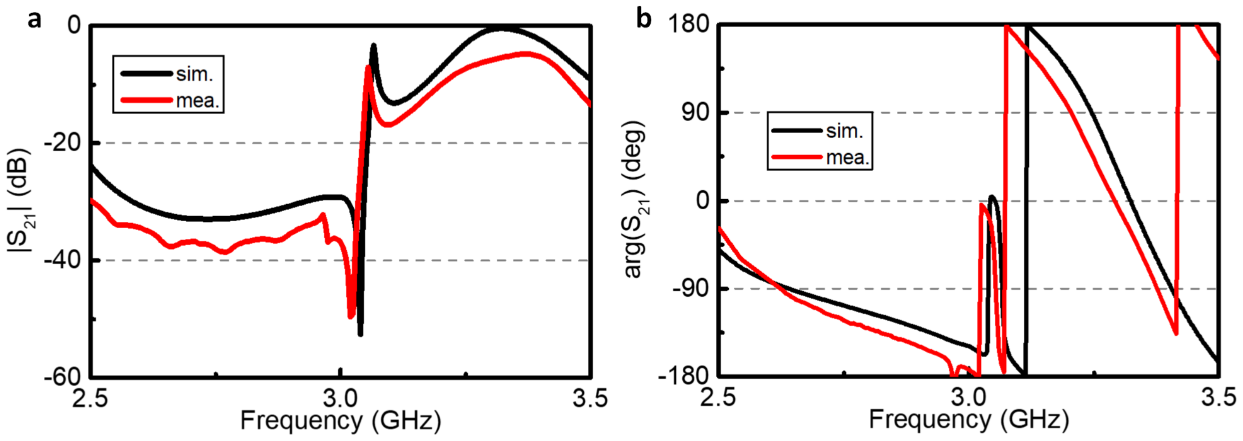

Supplementary Figure 5 | Simulated and measured transmission coefficients from Port 1 to Port 2 of the total structure: (a) simulated and measured magnitude of the transmission coefficient and **(b)** the simulated and measured phase of the transmission coefficient.

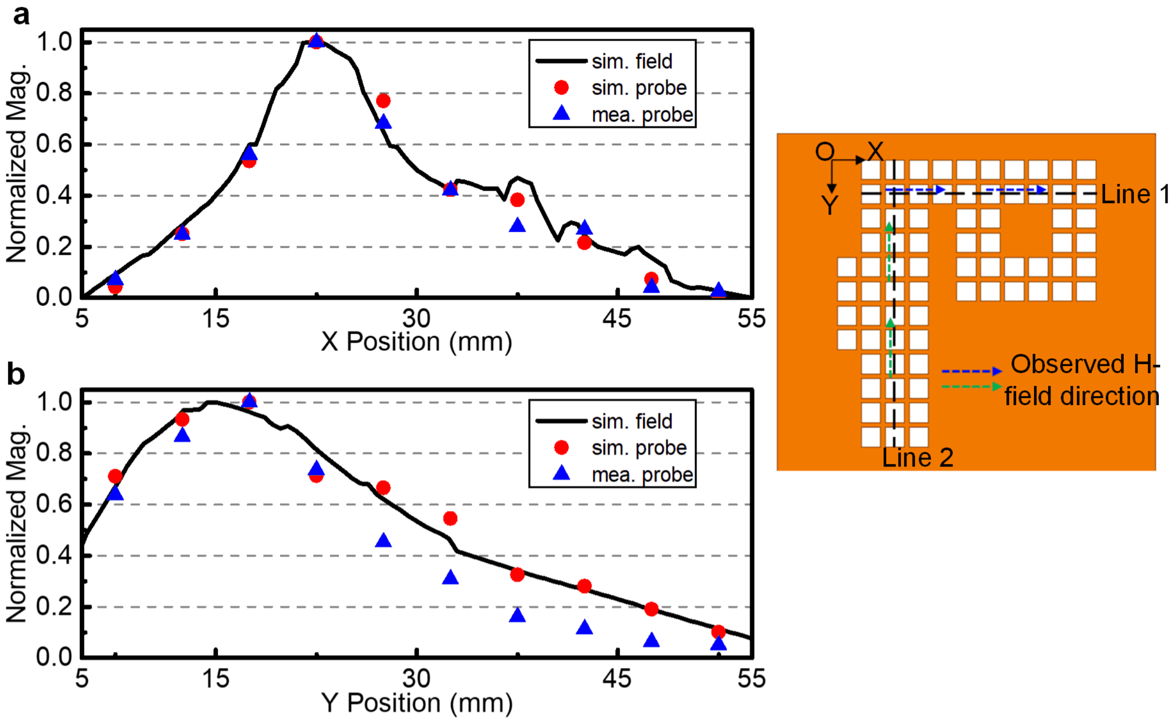

Supplementary Figure 6 | Comparisons between the exact field distribution and the transmission coefficients from input port to the probe: (a) The simulated x-component's magnitude of the surface magnetic field, simulated and measured magnitude of coupling coefficients between Port 1 and Port 3 along line 1 (blue) on which $y = 7.5mm$. **(b)** The simulated y-component's magnitude of the surface magnetic field, simulated and measured magnitude of coupling coefficients along line 2 (green) on which $x = 12.5mm$. All the data are normalized to the maximum, respectively.

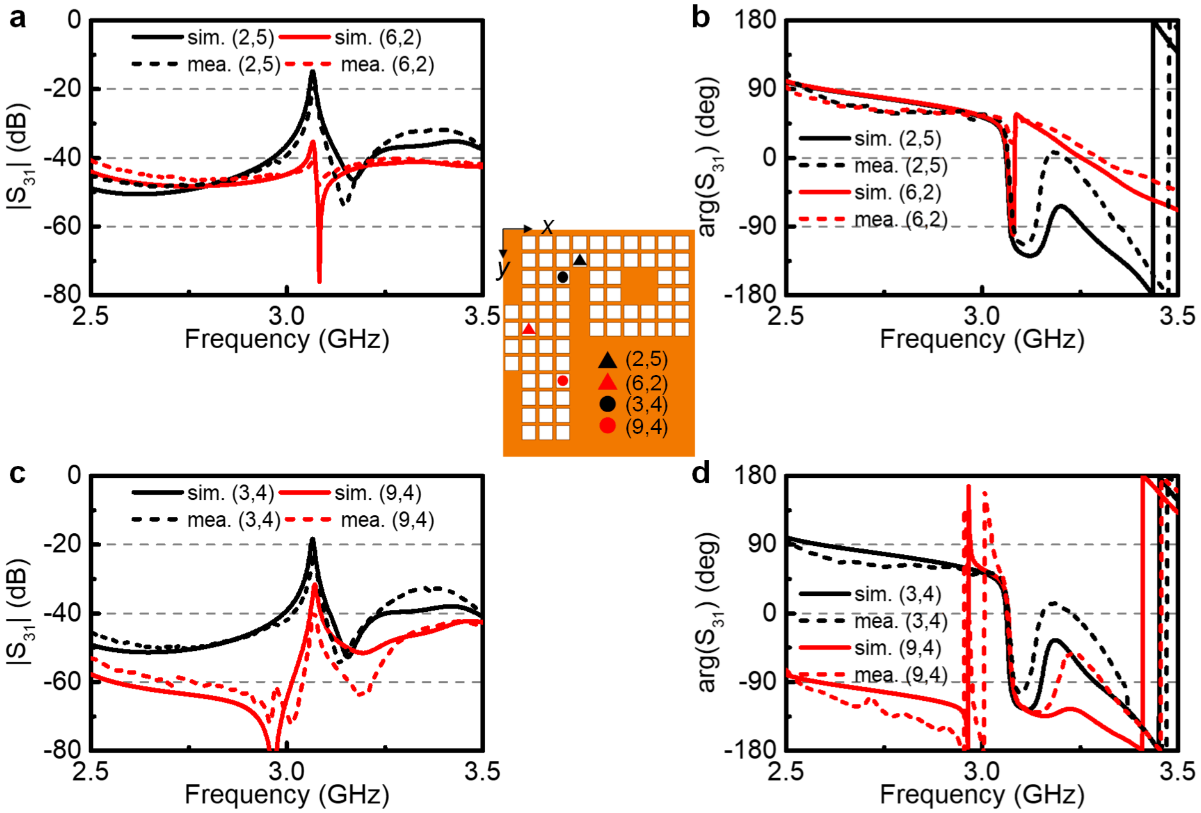

Supplementary Figure 7 | Comparisons between simulated and measured transmission coefficients at different points as an example: (a) and **(b)** simulated and measured magnitude and phase of the transmission coefficients from the input port to the probe at point (2,5) and (6,2). In these cases, the *x*-component of the surface magnetic field is measured. **(c)** and **(d)** simulated and measured magnitude and phase of the transmission coefficients at point (3,4) and (9,4). In these cases, the *y*-component of the surface magnetic field is measured.

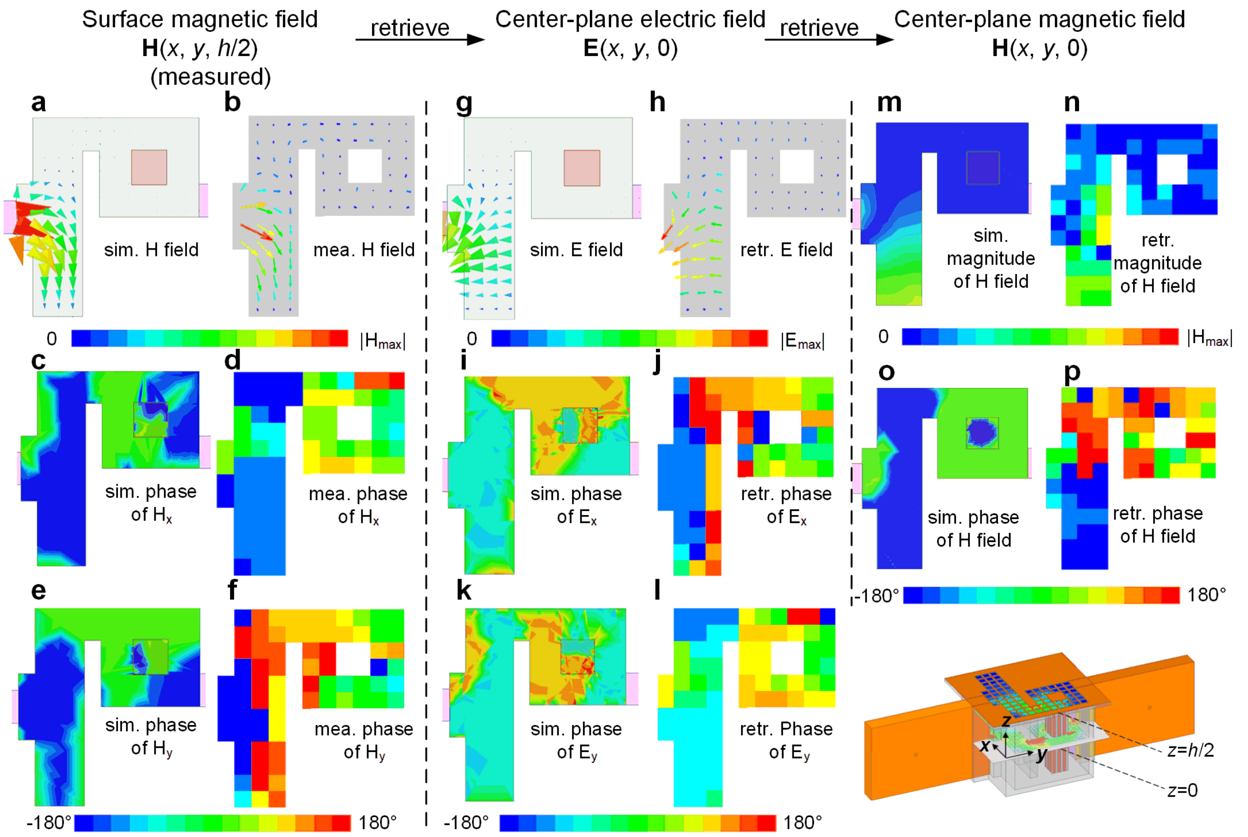

Supplementary Figure 8 | The simulated and measured or retrieved field distributions in each step of the normal medium with a dopant: (a) and **(b)** vector distribution of simulated and measured surface magnetic field, **(c)-(f)** phase distribution of simulated and measured surface magnetic field's x and y components. Notice that the red and blue color represent the same phase. **(g)** and **(h)** vector distribution of simulated and retrieved electric field on the center plane, **(i)-(l)** phase distribution of simulated and retrieved electric field's *x* and *y* components on the center plane, **(m)** and **(n)** magnitude of simulated and retrieved magnetic field on the center plane, **(o)** and **(p)** phase of simulated and retrieved magnetic field on the center plane. All the results are measured at 3.9 GHz.

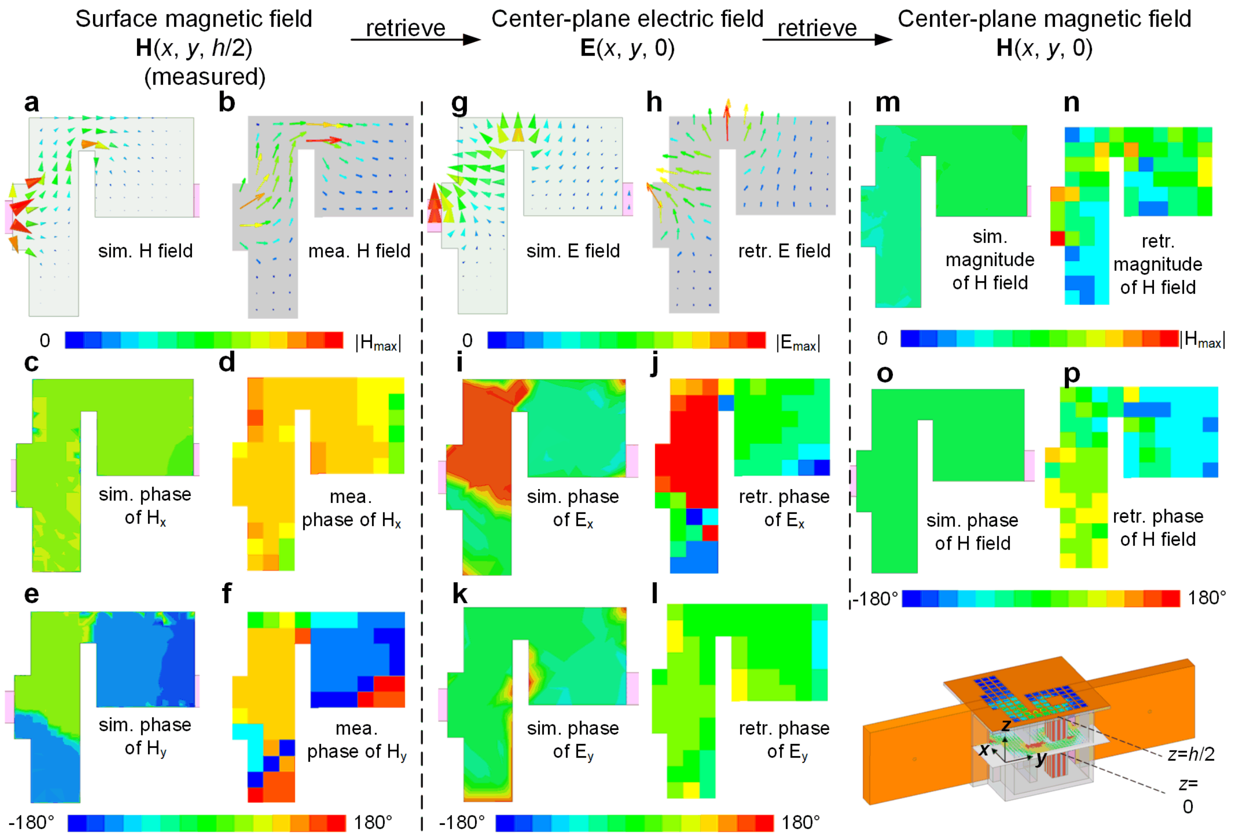

Supplementary Figure 9 | The simulated and measured or retrieved field distributions in each step of the undoped ENZ medium: (a) and **(b)** vector distribution of simulated and measured surface magnetic field, **(c)-(f)** phase distribution of simulated and measured surface magnetic field's x and y components, **(g)** and **(h)** vector distribution of simulated and retrieved electric field on the center plane, **(i)-(l)** phase distribution of simulated and retrieved electric field's *x* and *y* components on the center plane, **(m)** and **(n)** magnitude of simulated and retrieved magnetic field on the center plane, **(o)** and **(p)** phase of simulated and retrieved magnetic field on the center plane.

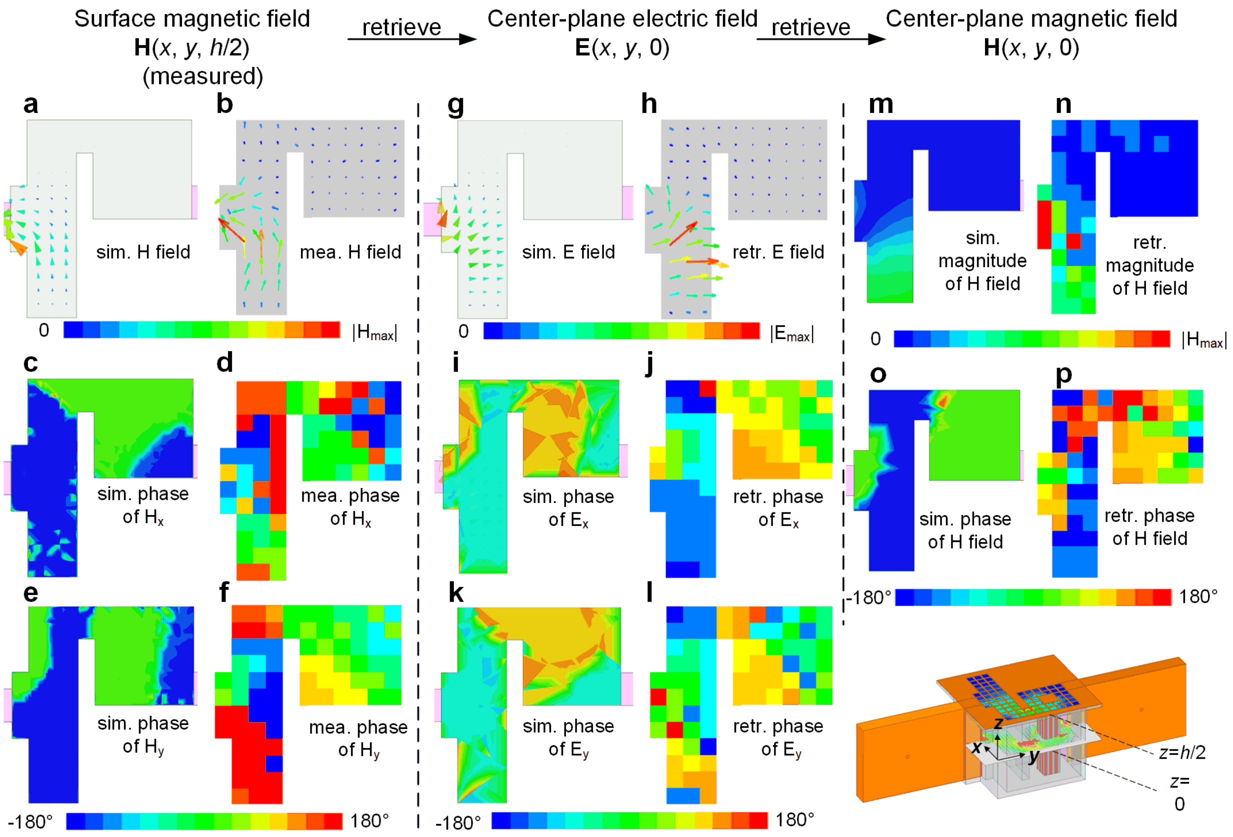

Supplementary Figure 10 | The simulated and measured or retrieved field distributions in each step of the normal medium without dopant: (a) and **(b)** vector distribution of simulated and measured surface magnetic field, **(c)-(f)** phase distribution of simulated and measured surface magnetic field's x and y components. Notice that the red and blue color represent the same phase. **(g)** and **(h)** vector distribution of simulated and retrieved electric field on the center plane, **(i)-(l)** phase distribution of simulated and retrieved electric field's *x* and *y* components on the center plane, **(m)** and **(n)** magnitude of simulated and retrieved magnetic field on the center plane, **(o)** and **(p)** phase of simulated and retrieved magnetic field on the center plane. All the results are measured at 3.9 GHz.

Supplementary Note 1. Detailed dimensions of the experimental platform's configuration.

The detailed geometry parameters of the experimental platform are labelled on the configuration shown in Supplementary figure 1. As depicted in Supplementary figure 1a, the whole structure contains an aluminum cavity with a 2-mm thick sidewall and a 50-mm height, which is about half the wavelength. The Γ-shaped cross section of this cavity is shown in Supplementary figure 1b. On its sidewall, two 10-mm thick slots are etched for the input and output waveguides. Each waveguide is composed by coating copper foils on a Teflon brick with dimensions of $50.0\times80.0\times10.0$ mm^3. For connections to the coaxial cables, two SMA connectors are soldered to the input and output waveguides, denoted as Port 1 and 2. Inside the cavity we place a dielectric block with a size of $11.0\times11.0\times50.0$ mm^3, on which 12 metal wires with widths of 1mm are printed for the suppression of degenerated resonances. This dielectric block, made of ceramics with a permittivity of 37.0, serves as a dopant embedded in ENZ media with a relative permittivity of 36.0. The geometry of the metal grid cover is also shown in Supplementary figure 1a, from which one can observe that the square hole's size is 4×4 mm^2 and the pitch in both x and y directions is 5 mm. In addition, Supplementary figure 1c depicts the detailed configuration of the B-field probe. On its top layer, a metal loop is printed with one end connected to a microstrip line and another to the ground by a metalized post. This probe is soldered to a semi-rigid coaxial cable, denoted as Port 3. The fabrication and assembling of this platform is explained in the Materials and Methods part specifically.

Supplementary Note 2. Numerical validation of the metal-grid-covered waveguide as an effective ENZ medium.

In this Supplementary Note, we numerically investigate the Poynting vector within both the proposed structure and in an actual ENZ medium. We have demonstrated in the main text that the metal grid cover serves as a PEC boundary approximately so that the cavity is equivalent to an ENZ medium[4]. Here a comparison between an ideal 2D ENZ medium and the proposed equivalent ENZ medium has been completed and plotted in Supplementary figure 2. Both cases share the same cross section and the same dopant. For the ideal ENZ medium used in Supplementary figure 2a

as a reference, we assume that there were no losses within this medium. The power flow on the center plane of the proposed configuration is exhibited in Supplementary figure 2b from which one can observe that although there may be leakage from the grid cover, the power flow resembles the ideal one well because the radiation loss out of the grid is relatively small. This ultra-low loss feature ensures both $\mathbf{n}\cdot\mathbf{S}_R=0$ and $\nabla\cdot\mathbf{S}_R=0$, making an analogy between the power flow and an inviscid and incompressible fluid. Since the vorticities do not exist neither, it is analogous to an ideal fluid. To further investigate the property of the metal grid cover, we make another comparison in Supplementary figure 3 between a waveguide with full metal cover and the proposed one with grid cover. The transmittance presented in Supplementary figure 3a demonstrates that a minor deterioration of 2.5 dB is introduced by the grid cover, decreasing the S_{21} parameter from -0.8 dB to -3.3 dB. We consider this reduction in transmittance inevitable for the reason that an open structure is required for the measuring system so the radiation loss is unavoidable. In addition, the grid cover serves as a reactive boundary so that there would also be a slight mode mismatch that increases the reflection. Nevertheless, the electromagnetic field distribution along with the Poynting vectors are barely affected according to Supplementary figures 3b and 3c, supporting that this system is capable of measuring the fields within an ENZ medium.

Supplementary Note 3. Derivation of Eq. (1) in the main text.

In this Supplementary Note, we derive the general mathematical expressions between the electromagnetic fields on the surface and center plane of a rectangular waveguide. To start with, we consider a waveguide with an arbitrary cross section in the *xy* plane and a height *h* in the *z* direction, as discussed in Fig. 1 and Supplementary figure 1. Under a TE_{10} incidence, no *z*-polarized electric fields are excited and the electric fields within the waveguide is consequently expressed as

$$\mathbf{E}(x,y,z) = E_x(x,y,z)\hat{\mathbf{x}} + E_y(x,y,z)\hat{\mathbf{y}} \tag{S1}$$

Due to the boundary conditions, the tangential electric fields at $z=h/2$ and $z=-h/2$ are zero, which can be written as

$$\mathbf{E}(x,y,h/2) = \mathbf{E}(x,y,-h/2) = 0\,. \tag{S2}$$

Since the electric field only has a non-zero value between $z=h/2$ and $z=-h/2$, it is reasonable to perform a Fourier transformation along the *z* direction so that the electric field can be expanded as a Fourier series

$$\mathbf{E}(x,y,z)=\sum_n \cos(\frac{n\pi z}{h})[E_x^{e,n}(x,y)\hat{\mathbf{x}}+E_y^{e,n}(x,y)\hat{\mathbf{y}}]+\sum_n \sin(\frac{2n\pi z}{h})[E_x^{o,n}(x,y)\hat{\mathbf{x}}+E_y^{o,n}(x,y)\hat{\mathbf{y}}] \quad \text{(S3)}$$

Notice that the waveguide is excited under the incidence of TE_{10} mode and we have an expression of the incident wave within the input waveguide that follows

$$\mathbf{E}^{\text{inc}}(x,y,z)=\cos(\frac{n\pi z}{h})E_0\hat{\mathbf{y}} \quad \text{(S4)}$$

The incidence is orthogonal to the higher even modes $E^{e,n}$ (n>1) and all the odd modes $E^{o,n}$. Consequently, only the basic mode $E^{e,1}$ is excited in the waveguide as LSE^z_{10} mode and we can rewrite the electric fields in the waveguide as

$$\mathbf{E}(x,y,z)=\cos(\frac{\pi z}{h})[E_x(x,y)\hat{\mathbf{x}}+E_y(x,y)\hat{\mathbf{y}}] \quad \text{(S5)}$$

This equation analytically describes the electric fields at any locations with relations to the center plane. Moreover, when applying the Faraday's law $\nabla\times\mathbf{E}=i\omega\mu\mathbf{H}$ to Eq. (S5), the magnetic field within the waveguide is calculated to be

$$\mathbf{H}=\frac{1}{i\omega\mu_0}\cos(\frac{\pi z}{h})[-\frac{\partial E_x(x,y)}{\partial y}+\frac{\partial E_y(x,y)}{\partial x}]\hat{\mathbf{z}}-\frac{\pi}{i\omega\mu_0 h}\sin(\frac{\pi z}{h})\hat{\mathbf{z}}\times[E_x(x,y)\hat{\mathbf{x}}+E_y(x,y)\hat{\mathbf{y}}] \quad \text{(S6)}$$

On the surface z=-h/2, the z component of the magnetic field is zero according to Eq. (S6) and a simpler form of Eq. (S6) is written as

$$\begin{cases} H_x(x,y,-h/2)=-\dfrac{\pi}{i\omega\mu_0 h}E_y(x,y) \\ H_y(x,y,-h/2)=\dfrac{\pi}{i\omega\mu_0 h}E_x(x,y) \end{cases} \quad \text{(S7)}$$

which is equivalent to Eq. (1) within the main text.

Supplementary Note 4. The measurement system and additional measured results for validation.

Photographs of the measuring system including the experimental process and the detailed structure of the components are all shown in Supplementary figure 4. In Supplementary figure 4a the entire measuring system is pictured while the photos of the assembled experimental platform and the *B*-field probe are both shown in Supplementary figures 4b and 4c. To begin with, we first measured the transmittance from the input waveguide to output, denoted as S_{21}. The simulated and measured transmittance is depicted in Supplementary figure 5, in which a total reflection at 3.01 GHz

and a supercoupling at 3.06 GHz are both observed, corresponding to the doped ENZ medium's property[8]. The measured peak transmittance is -5.6 dB, which is about 2.3 dB lower than the simulated one mainly due to fabrication errors and a higher dielectric loss of the dopant. Furthermore, the metal grid cannot maintain a perfect planar geometry when screwed with the cavity, resulting in a varying height of the cavity and a minor frequency shift of the transmission peak.

When measuring the magnetic fields on the surface, we connect the input port (Port 1) and the *B*-field probe (Port 3) to the vector network analyzer, as shown in Supplementary figure 4a. In this situation, a 50-Ohm load is connected to the output Port 2 to achieve a perfect absorption. We adopted a laser aligning system in addition to the original experimental platform as depicted in Supplementary figure 4. The alignment method ensures a constant penetration depth and a similar location for each window. We put the experimental platform between a laser emitter and a ruler standing on the optical table. Before the measurement process of each column, we make sure that the laser propagates exactly over the middle line of each square window. In terms of z axis, the light spot on the ruler reads 52 mm, which is the height of the probe when the penetration depth is exactly 2 mm. In this case, we can ensure that the probe locates exactly at the middle of the window while the penetration depth is always 2 mm as long as the laser is illuminating on the probe. In this case, the measured S_{31} is strictly proportional to the local magnetic field. Here a validation is provided in Supplementary figure 6 by comparing the normalized magnitudes of the simulated magnetic fields, simulated S_{31} and the measured data. Supplementary figure 6a shows these three quantities with variation *s* to *x* locations along line 1 on the second row at 3.06 GHz. Both the measured and simulated S_{31} parameters agree well and maintains a proportion with the simulated fields. For the *y* direction, we plot them in Supplementary figure 6b.

Furthermore, here we show that the simulated and measured spectra of S_{31} agree very well. As depicted in Supplementary figure 7, we investigate the S_{31} at four different locations. The coordinates (y_0, x_0) refers to the center of the hole in y_0-th row, x_0-th column. In Supplementary figures 7a and 7b, the *x*-polarized magnetic fields at the two points labelled by triangles are measured by putting the probe in *yz* plane and shown together with the simulations. For the points labelled by dots, the *y*-polarized magnetic fields are measured using similar method and compared with the simulated results in Supplementary figures 7c and 7d. In every cases the experimental results match well with the numerical ones.

Supplementary Note 5. The measured electromagnetic fields for undoped ENZ medium or normal media with or without dopants.

In the main text, we deal with the full measurement and retrieving process of the doped ENZ case, which is of the main focus of this paper. Here, in this Supplementary Note and Supplementary figures 8-10, the intermediate results of the electromagnetic fields of the three other cases are presented and discussed. Supplementary figure 8 shows the electromagnetic fields measured at 3.9 GHz. According to Supplementary figure 8, a resonance is excited in the corner for the electromagnetic fields only exist there, offering an explanation to the blockage by the obstacle. The simulated and measured or retrieved fields in this region match each other well. However, in other areas where the magnitude is relatively small, the measured and retrieved phase distribution are not so accurate. Nevertheless, the imaged Poynting vectors, as shown in Fig. 4 of the main text, proves that a vortex exists in the normal medium and supports that the irrotational power flow is a characteristic feature of NZI medium as demonstrated in ref. (*31*) and the main text. The measured and retrieved field distributions at either 3.06 GHz or 3.9 GHz are depicted in Supplementary figures 9 and 10 as the reference results for undoped media. In Fig. S9, the simulated and measured surface magnetic fields, together with the electric fields and magnetic fields on the center plane at 3.06 GHz are shown in the first, second, and third columns. Agreements are noted between the simulated and measured results both the vector and phase distributions. From the third column, one can observe that the magnetic fields on the center plane remains a constant magnitude and phase distribution, corresponding to the ENZ property.

References

1. I. Liberal and N. Engheta, Near-zero refractive index photonics, *Nat. Photonics*, **11**, 149–158 (2017).
2. M. Silveirinha and N. Engheta, Tunneling of electromagnetic energy through subwavelength channels and bends using ε-near-zero materials, *Phys. Rev. Lett.* **97**, 157403, (2006).
3. H. Feng Ma, J. Hui Shi, Q. Cheng, and T. Jun Cui, Experimental verification of supercoupling and cloaking using mu-near-zero materials based on a waveguide," *Appl. Phys. Lett.* **103**, 021908, (2013).
4. B. Edwards, A. Alù, M. E. Young, M. Silveirinha, and N. Engheta, Experimental Verification of Epsilon-Near-Zero Metamaterial Coupling and Energy Squeezing Using a Microwave Waveguide, *Phys. Rev. Lett.*, **100**, 033903 (2008).

5. R. Liu, Q. Cheng. T. Hand, J. J. Mock, T. J. Cui, S. A. Cummer, and David R. Smith, Experimental Demonstration of Electromagnetic Tunneling Through an Epsilon-Near-Zero Metamaterial at Microwave Frequencies, *Phys. Rev. Lett.*, **100**, 023903 (2008).
6. M. Mitrovic, B. Jokanovic, and N. Vojnovic, Wideband tuning of the tunneling frequency in a narrowed epsilon-near-zero channel, *IEEE Antennas Wireless Propag. Lett.*, **12**, 631–634 (2013).
7. I. Liberal, A. M. Mahmoud, and N. Engheta, Geometry-invariant resonant cavities, *Nat. Commun.*, **7**, 10989 (2016).
8. I. Liberal, A. M. Mahmoud, Y. Li, B. Edwards, and N. Engheta, Photonic doping of epsilon-near-zero media, *Science*, **355**, 1058–1062 (2017).
9. J. Luo, B. Liu, Z. H. Hang, Y. Lai, Coherent perfect absorption via photonic doping of zero-index media. *Laser Photonics Rev.* **12**, 1800001 (2018).
10. Z. Zhou, Y. Li, H. Li, W. Sun, I. Liberal, and N. Engheta, Substrate-integrated photonic doping for near-zero-index devices, *Nat. Commun.*, **10**, 4132, (2019).
11. Z. Zhou and Y. Li, "N-Port Equal/Unequal-Split Power Dividers Using Epsilon-Near-Zero Metamaterials," *IEEE Trans. Microw. Theory Techn*, **69**, 1529-1537 (2021).
12. I. Liberal and N. Engheta, Manipulating thermal emission with spatially static fluctuating fields in arbitrarily shaped epsilon-near-zero bodies, *PNAS*, **115**, 2878-2883 (2018).
13. S. Enoch, G. Tayeb, P. Sabouroux, N. Guérin, and P. Vincent, A metamaterial for directive emission, *Phys. Rev. Lett.* **89**, 213902 (2002).
14. E. Forati, G. W. Hanson and D. F. Sievenpiper, An Epsilon-Near-Zero Total-Internal-Reflection Metamaterial Antenna, *IEEE Trans. Antennas Propag.*, **63**, 1909-1916 (2015).
15. M. Memarian and G. Eleftheriades, "Dirac leaky-wave antennas for continuous beam scanning from photonic crystals." *Nat. Commun.* **6**, 5855 (2015).
16. Z. Zhou and Y. Li, A Photonic-Doping-Inspired SIW Antenna With Length-Invariant Operating Frequency, *IEEE Trans. Antennas Propag.*, **68**, 5151-5158 (2020).
17. M. Navarro-Cía, M. Beruete, I. Campillo, and M. Sorolla, Enhanced lens by ε and μ near-zero metamaterial boosted by extraordinary optical transmission, *Phys. Rev. B* **83**, 115112 (2011).
18. M. Navarro-Cía, M. Beruete, M. Sorolla, and N. Engheta, Lensing system and Fourier transformation using epsilon-near-zero metamaterials, *Phys. Rev. B* **86**, 165130 (2012).
19. J. C. Soric and A. Alù, Longitudinally Independent Matching and Arbitrary Wave Patterning Using ε-Near-Zero Channels, *IEEE Trans. Microw. Theory Techn.*, **63**, 3558-3567 (2015).
20. M. Z. Alam, I. De Leon and R. W. Boyd, Large optical nonlinearity of indium tin oxide in its epsilon-near-zero region, *Science* **352**, 795-797 (2016).
21. E. G. Carnemolla, L. Caspani, C. DeVault, M. Clerici, S. Vezzoli, V. Bruno, V. M. Shalaev, D. Faccio, A. Boltasseva, and M. Ferrera, Degenerate optical nonlinear enhancement in epsilon-near-zero transparent conducting oxides, *Opt. Mater. Express* **8**, 3392-3400 (2018)
22. A. Capretti, Y. Wang, N. Engheta, and L. D. Negro, Comparative Study of Second-Harmonic Generation from

Epsilon-Near-Zero Indium Tin Oxide and Titanium Nitride Nanolayers Excited in the Near-Infrared Spectral Range, *ACS Photonics* **2**, 1584–1591 (2015).

23. N. Kinsey, C. DeVault, J. Kim, M. Ferrera, V. M. Shalaev, and A. Boltasseva, Epsilon-near-zero Al-doped ZnO for ultrafast switching at telecom wavelengths, *Optica* **2**, 616-622 (2015).
24. O. Reshef, I. De Leon, M. Z. Alam, and R. W. Boyd, Nonlinear optical effects in epsilon-near-zero media, *Nat. Rev. Mater.*, **4,** 535–551 (2019).
25. R. W. Ziolkowski, Propagation in and scattering from a matched metamaterial having a zero index of refraction, *Phys. Rev. E*, **70**, 046608 (2004).
26. P. Moitra, Y. Yang, Z. Anderson, I. I. Kravchenko, D. P. Briggs, and J. Valentine, Realization of an all-dielectric zero-index optical metamaterial, *Nat. Photonics*, **7**, 791–795 (2013).
27. Y. Li, S. Kita, P. Muñoz, O. Reshef, D. I. Vulis, M. Yin, M. Lončar, and E. Mazur, On-chip zero-index metamaterials, *Nat. Photonics*, **9**, 738–742 (2015).
28. B. Edwards, A. Alù, M. G. Silveirinha, and N. Engheta, Experimental Verification of Plasmonic Cloaking at Microwave Frequencies with Metamaterials, *Phys. Rev. Lett.* **103**, 153901 (2009).
29. O. Reshef, P. Camayd-munoz, D. I. Vulis, Y. Li, M. Loncar, and E. Mazur, Direct Observation of Phase-Free Propagation in a Silicon Waveguide, *ACS Photonics* **4**, 2385−2389 (2017).
30. J. So, G. Yuan, C. Soci, and N. I. Zheludev, Enhancement of luminescence of quantum emitters in epsilon-near-zero waveguides, *Appl. Phys. Lett.* **117**, 181104 (2020)
31. I. Liberal, M. Lobet, Y. Li, and N. Engheta, Near-zero-index media as electromagnetic ideal fluids, *PNAS*, **117**, 24050-24054 (2020).
32. See Supplementary Materials.
33. P. R. West, et al. Searching for better plasmonic materials, *Laser Photonics Rev.* **4**, 795-808 (2010).
34. G. Naik, J. Kim, and A. Boltasseva, Oxides and Nitrides as Alternative Plasmonic Materials in the Optical Range, *Opt. Mater. Express* **1**, 1090–1099 (2011).
35. M. Ordal, R. Bell, R. Alexander, Jr, L. Long and M. Querry, Optical properties of fourteen metals in the infrared and far infrared: Al, Co, Cu, Au, Fe, Pb, Mo, Ni, Pd, Pt, Ag, Ti, V, and W, *Appl. Opt.*, **24**, 4493, (1985).
36. F. M. White, *Fluid Mechanics*, McGraw-Hill Education, (2015).
